# Green synthesis and characterization of silver nanoparticles from Gilaburu under varying temperature and pH, with antimicrobial activity and spectral inconsistencies

**DOI:** 10.1038/s41598-025-00595-1

**Published:** 2025-11-04

**Authors:** Serap Güneş, Demet Ektiren, Hasan Vardin

**Affiliations:** 1 Diyarbakır Agricultural Vocational School, Food Processing Department, Dicle University, Diyarbakır, Turkey; 2 Engineering Faculty, Food Engineering Department, Harran University, Şanlıurfa, Turkey; 3 Diyarbakır Agricultural Vocational School, Food Processing Department, Dicle University, Diyarbakir, Turkey

**Keywords:** Antibacterial activity, Green synthesis, Silver nanoparticles, *Viburnum opulus*, Biotechnology, Chemical biology, Microbiology, Plant sciences, Nanoscience and technology

## Abstract

The wild-grown Gilaburu (*Viburnum opulus L.*) fruit, traditionally consumed in Turkey and some Central and Northern European countries, has remarkable bioactive properties due to its high anthocyanin, phenolic compound, and organic acid content. This study produced silver nanoparticles (AgNPs) from Gilaburu fruit using a reduction-based biosynthetic approach with plant extract as a green synthesis method. The research aimed to determine the optimum AgNP production parameters under different temperatures (50 °C, 90 °C, and 100 °C) and pH (7, 8, 9, 10, and 11) conditions and to evaluate the antimicrobial properties of these nanoparticles. The characterization of the synthesized AgNPs was carried out using UV-Vis spectroscopy, Fourier transform infrared spectroscopy (FTIR), Scanning Electron Microscopy–Energy Dispersive Spectroscopy (SEM-EDX), and X-Ray Diffraction (XRD) techniques. The size of the obtained nanoparticles varied between 20.25 and 25.24 nm on average, depending on each synthesis condition. Antimicrobial activity assessment was performed using the standard agar diffusion method. Four bacterial strains (*S. aureus*,* E. coli*,* B. subtilis*,* P. aeruginosa*) and one yeast strain (*C. albicans*) were used. Silver nanoparticle suspensions were applied to the agar surface, and the inhibition zones formed after the incubation period were measured to evaluate their antimicrobial effects. Synthesized AgNPs showed antibacterial activity against *E. coli*,* S. aureus*, and *B. subtilis*; however, no antimicrobial activity was detected against *P. aeruginosa* and *C. albicans*. Spectral analysis results of some samples that were not completely crystalline were similar to those of crystalline AgNPs.

## Introduction

Nanotechnology is a branch of scientific studies that aims to create new structures at the nanoscale (ranging from 1 to 100 nm in diameter) by controlling materials at the atomic and molecular level^1,2^ The unique properties and potent mechanisms of action of nanoparticles create a need for continued innovation in areas such as global food security and many other areas^3–5^With the recent developments in the field of nanoscience, the applications of metallic nanoparticles have spread to a wide range. In this context, it is known that various types of metal NPs, such as Silver (Ag), Gold (Au), Copper (Cu), Cadmium (Cd), Titanium (Ti), Zinc, and Iron oxide, are used effectively^6–8^ Two main methods produce nanoparticles: ‘’top-down’’ and ‘’bottom-up’’^9^. The top-down approach is a destructive process whereby large material is broken down into small pieces (from micro-sized to nanoparticles). The bottom-up approach is a constructive method wherein nanoparticles are synthesized by the assembly of individual atoms (ions) or molecules^10^Many classical methods have been applied to produce nanoparticles in solution for a long time^11^. Although it is possible to synthesize nanoparticles in the desired size and morphology with classical synthesis methods, due to the disadvantages of these methods, more economical, simple, and non-toxic methods are being investigated with green nanotechnology and for this reason, “Green nanotechnology”, which includes cheaper, environmentally friendly, non-toxic biological methods, has come to the fore^12^. Enzymes, microorganisms, fungi, and plant extracts are used in production^13^. Components in plant extracts contribute significantly to the production processes. Some conditions during production significantly affect the structure of the produced nanoparticles. Some of these conditions are; temperature, pH, reaction solution time, and metal salt concentration^13^. Nowadays, nanoparticles are synthesized from different parts of plant sources because they are naturally occurring, fast-forming, cheap, and environmentally friendly. Green synthesis consists of a solution containing plant extract and metal ions^14^. Plant extracts provide a medium to reduce metal ions. It also acts as a coating agent to maintain the synthesized NPs. Plants are rich in flavonoids, phenolics, proteins, amino acids, vitamins, polysaccharides, and alkaloids. These components are stabilizers and reductants for nanoparticle synthesis^15,16^. These components facilitate metal ions’ cost-effective and safe reduction^17^. Biosynthesized AgNPs have been reported to have numerous pharmacological effects, including cytotoxic, anticancer, antifungal, antibacterial, and antioxidant properties^18–22^.

The spread of alien plant species can cause significant structural and functional changes in the local flora due to climate change and various environmental factors. These species increase their spread by adapting to the climatic and topographic characteristics of the ecosystems they are located in and create multifaceted effects such as competition for resources, transformation in vegetation, genetic pollution, and disruptions in ecosystem functioning^23–29^ *Viburnum opulus* is a plant that prefers moist soils rich in organic matter and has a high water requirement. It requires sunny areas for optimum fruit quality and yield. For this reason, it grows naturally mostly on forest edges, in sparsely wooded areas, and in areas close to water sources. According to some sources, its homeland is reported as Turkey, and according to others as Central China, and it has a high ability to adapt to continental climate conditions. Although the species originates from Asia and Europe, it is cultivated as an ornamental plant in a wide geography such as Turkestan, Siberia, America, Europe, North Asia, and North Africa. In Turkey, it is distributed naturally in many provinces, especially in the Central Anatolia and Black Sea Regions, especially in Kayseri and Yozgat^30^.In Turkey, known as the “European cranberry bush,” Gilaburu is a member of the Adoxaceae family. It is also known by various names, such as the “snowball tree, European cranberry bush, Cherry tree, and Crank bark” in Northern Asia, Africa, and Eastern Europe. Sometimes resembling a bush or a stunted tree, the Gilaburu can grow up to 4 m tall, and its red fruits are edible^31–35^ *Viburnum opulus L.* contains high degrees of bioactive components such as catechins, phenolic compounds, anthocyanins, chlorogenic acids, and quercetin^36,37^. Due to its rich bioactive content, the fruit treats many diseases such as stomach, heart, kidney, lung, and tuberculosis. In addition, its fruits and flowers are used in traditional medicine as a laxative, sedative for vascular spasms and nervous disorders. The seeds and peel of this fruit, which has a cough-inhibiting effect, are used in Europe for the same purposes^35,38,39^.

We chose Gilaburu fruit for our study because it contains many bioactive components that reduce silver ions and provide stabilization after synthesis. It has been previously reported that the bioactive components found in *Viburnum opulus* have antimicrobial and anti-inflammatory effects^40,41^ Despite their high nutritional value and functional properties, Gilaburu (*Viburnum opulus L*.) fruits are underutilized because they do not receive the attention, they deserve in the food industry or functional food products^42^. Gilaburu (*Viburnum opulus L*.) can play an important role in organic food systems. Developing new products and innovative value chains, especially in the context of healthy, sustainable, and organic foods, are promising strategies. This approach can create economic and sustainable benefits for the consumer from production^43^. Gilaburu can be strategically important in these areas due to its health benefits and environmental sensitivity. This fruit is considered to have great potential in terms of sustainability. Gilaburu (*Viburnum opulus L*.) fruit has a powerful antioxidant effect due to the wide variety of phytochemicals it contains^44–48^. Thanks to these properties, Gilaburu expresses its capacity to neutralize free radicals, which is important for health. Therefore, *Viburnum opulus* fruit can be used to reduce silver ions in AgNP production. Silver nanoparticles are highly preferred in many sectors due to their physicochemical and antibacterial properties. The antibacterial properties of silver nanoparticles are the most important elements. Various factors affect the size of nanoparticles during production. Some of these factors are the pH and temperature of the medium, the duration of the reaction, and the reducing substances. Synthesis via plant is less toxic and more environmentally friendly than other methods^49,50^. The main goal of our study is to regulate how nanoparticles are formed and to control this process within the framework of a certain mechanism. This is a very important issue because factors such as nanoparticle size, shape, and chemical properties can vary depending on the production mechanism. Silver nanoparticles (AgNPs) were produced using *Viburnum opulus L.* extract, a biological source. Particularly biological methods are important because they are generally environmentally friendly and do not require toxic chemicals. In our study, the effects of variables such as temperature and pH on nanoparticle formation were discussed in detail in the production of nanoparticles from Gilaburu (*Viburnum opulus L*.) extract. Thus, we believe that by understanding these processes in depth, we have paved the way for producing more controlled nanoparticles with desired properties.

## Materials and methods

### Gilaburu plant material

**Fig. 1:**
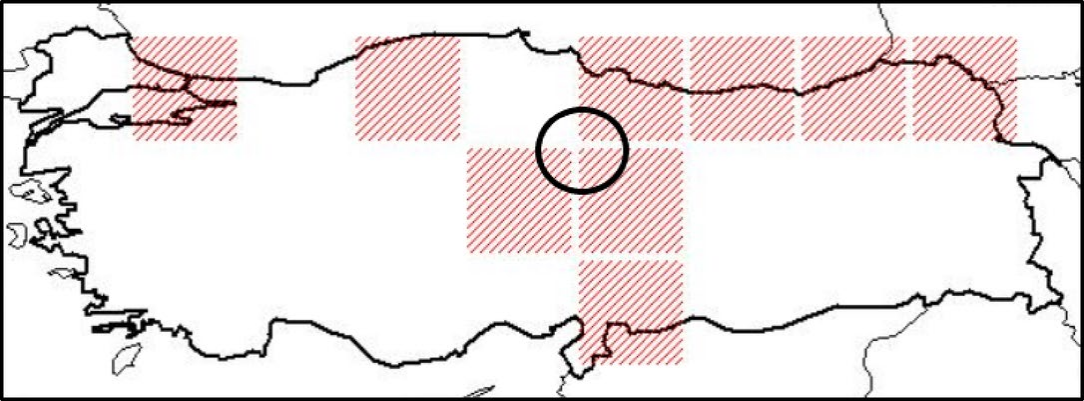
Map showing the geographical distribution of sampling areas of *Viburnum opulus*.

Gilaburu (*Viburnum opulus L.*) fruits were collected from 22 different regions of Yozgat province in the Central Anatolia Region of Turkey, which are not cultivated and are self-growing. These regions were selected to examine the natural diversity and quality characteristics of Gilaburu fruit, reflecting the different climate characteristics of Yozgat. The areas where samples of the *Viburnum opulu*s species were collected were geographically mapped based on coordinate data obtained from the Turkish Plants Data Service (TÜBİVES) and these areas are shown in Fig. 1.

### Chemicals

All chemicals used in this study were of analytical grade and were commercially supplied. Silver nitrate (AgNO₃, ≥ 99.9%, Merck) and plant extract prepared from Gilaburu (*Viburnum opulus L*.) fruit in laboratory conditions were used in the synthesis of silver nanoparticles. Deionized water was used in the preparation of solutions and washing processes. Sodium hydroxide (NaOH, ≥ 98%, Merck) and hydrochloric acid (HCl, 37%, Merck) solutions were used for pH adjustments, when necessary. Potassium bromide (KBr, ≥ 99%, Merck) was used to pellet the samples in FTIR analyses. Mueller Hinton Agar and Tryptic Soy Agar (Merck) media were prepared in the evaluation of antimicrobial activities; four bacterial strains and one yeast strain were used in the ATCC standard. Bacterial and fungal strains were obtained from a private laboratory. Solutions were prepared fresh in distilled water.

### Preparation of extract from *Viburnum opulus L.* fruit and silver nitrate (Agno₃) solution

The samples were cleaned by washing them with drinking water and then with distilled water, and the moisture was removed by drying them with filter paper. The dried samples were stored at -20 °C until use. The samples used in the analysis were pureed using a laboratory-scale grinder before use. The fruit extract was prepared by modifying the method of David et al.^51^. 10 g of ground and pureed whole fruit was suspended with 200 ml of pure water in a bottle to obtain the extract^51^. The resulting mixture was kept in a shaking incubator at 37 °C by shaking. At the end of the specified time, the plant extract was passed through filtration paper, and the clear aqueous solution was kept at 4 °C.1 mM AgNO₃ nanoparticle solution was prepared by mixing the specified amount of solid AgNO₃ nanoparticle in pure water with a magnetic stirrer. The solution was stored in the dark for further studies.

### Synthesis of silver nanoparticles at different pH and temperature conditions

**Fig. 2:**
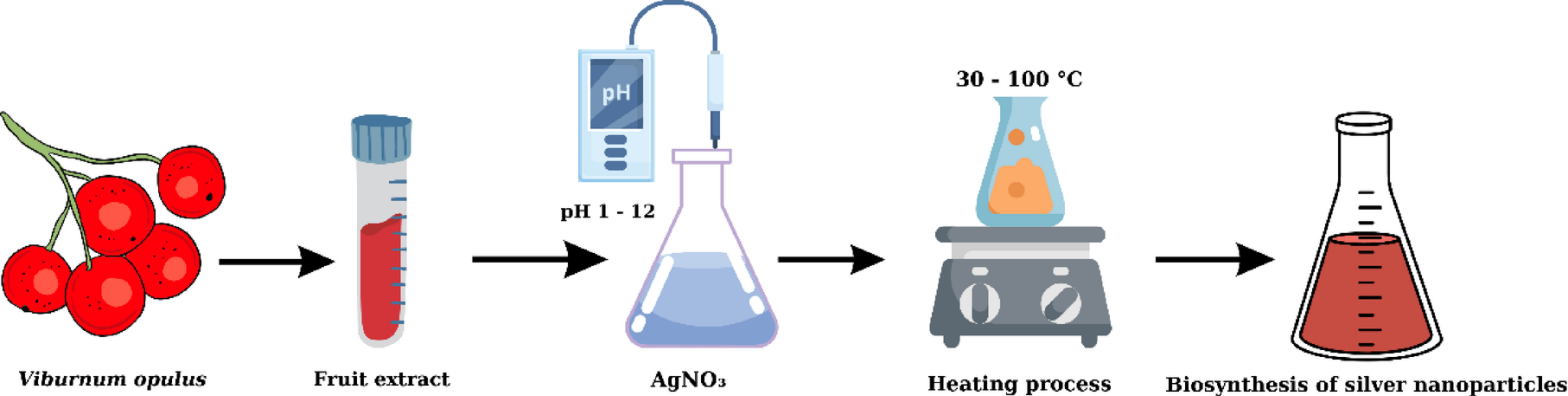
Time-dependent color change of AgNPs synthesized from *Viburnum opulus* extract.

Silver nanoparticles were produced using *Viburnum opulus L.* extract, which is rich in bioactive substances that reduce silver ions. Before mixing *Viburnum opulus* extract with AgNO₃, the pH degrees of silver nitrate, which was a known initial concentration in the synthesis medium, was adjusted to 1, 2, 3, 4, 5, 6, 7, 8, 9, 10, 11 and 12, respectively. 10 ml of Gilaburu extract was mixed with 50 ml of pH-adjusted 1 mM concentration silver nitrate solution. To investigate the formation of silver nanoparticles, mixtures prepared at eight different temperatures (30, 40, 50, 60, 70, 80, 90, and 100 °C) and twelve different pH concentrations were kept in a dark environment for a certain period (0–24 h). As a result of systematic observation, the solution color change was observed. The color changed from light yellow to brown depending on the ambient temperature and pH concentration. The reaction development process is given in Fig. 2. This change confirmed that silver ions were reduced and silver nanoparticles were formed. All reactions were started simultaneously. The corresponding reaction time was completed in the shortest possible time and all were stopped together.

### Characterization of synthesized AgNPs (silver nanoparticles)

The morphologies, stabilities, size distributions, functional parts, and crystal structures of AgNPs synthesized under different conditions from *Viburnum opulus* fruit extract were characterized. The brownish color in the solution was observed due to the interaction of *Viburnum opulus L.* extracts with a silver metal salt, which confirmed the synthesis of AgNPs. UV-Vis, FTIR, XRD, and SEM-EDX were utilized for the characterization of AgNPs^52–54^.

### Ultraviolet-visible spectrophotometry (UV- Vis)

A UV-Vis spectrum of silver nanoparticles analyzed in the ultraviolet-visible spectrum was recorded in wavelength range from 300 nm to 900 nm^55,56^. The spectra of AgNPs were obtained by UV-Vis (Shimadzu, UV-128, UV-Vis Spectrophotometer, Japan) spectrophotometer.

### Scanning electron microscopy (SEM) with EDX analysis [SEM evaluation of AgNPs

SEM evaluation is typically used to determine the size of the produced nanomaterials and predict their morphological properties^57,58^. The samples used in the study were mounted on a metal carrier surface using double-sided conductive tapes and carbon-based paste. The samples were coated with gold/palladium (Au/Pd) alloy in a sputter coating unit to make them conductive. These coated samples were examined with a Zeiss Evo 50 (Germany) model scanning electron microscope (SEM) to perform morphological analyses. In addition, analyses were performed using energy dispersive X-ray spectroscopy (EDX) integrated with the SEM system to determine the elemental compositions of the samples.

### Fourier transform infrared spectroscopy (F-TIR)

The measurement made on an FTIR device examines the presence of different functional groups that help reduce silver ions. These functional groups play a role in lowering and stabilizing silver ions. The FTIR spectrum of AgNPs was measured in the wavelength range of 4000–500 cm‾¹^59,60^ FTIR (IRTracer-100 Shimadzu, Japan) was used to detect different functional groups on AgNPs.

### X-ray diffraction (XRD)

An XRD analyzes the synthesized AgNPs and provides information about the crystal structure size, phase composition, and interatomic distances. XRD analysis was performed using an X-ray diffractometer (XRD) (Bruker D8 Discover, Japan) using Cu-Kα radiation at 40 kV and 30 mA, θ to 2θ configuration. Spectral measurements were performed in the angular range of 5°–90° (2θ/min)^61^. The crystal size of AgNPs were characterized using the Debye-Scherrer formula.

$${\text{D}}\,=\,{\text{K}}\lambda /\beta {\text{cos}}\theta$$

D: crystal size, K: shape factor (about 0.9 for spherical particles), λ: wavelength of X-ray radiation used, β: full width at half maximum of the diffraction peak (in radians), θ: Bragg diffraction angle for the plane of interest.

### Antimicrobial activity

The antimicrobial effects of AgNPs obtained from the extract were tested against specific pathogens using the agar diffusion method. Four bacterial strains (ATCC standard bacterial culture) and one fungus were used in the study. Antimicrobial activities of silver nanoparticles were tested in defiance of *P. aeruginosa* and *E. coli*, *S. aureus* and *S. pyogenes*, and the fungus *C. albicans*. First, microorganisms were cultured in MHB and kept in an incubator at 37 °C for approximately 24 h. Then, bacteria were diluted in 0.9% saline equivalent to 0.5 McFarland standard. 100 µl of the revived strain culture was spread on MHA for bacteria and PDA for fungi petri dishes. Four wells (well diameter 6 mm) were created in each plate using a piercer. Then, 100 µl of AgNPs were added to each well. *E. coli*,* P. aeruginosa*,* S. pyogenes*,* S. aureus*, and *C. albicans* plates were kept in a 37 °C incubator for 24–48 h. At the end of the process, inhibition zone areas (mm) were measured in three directions using a digital caliper, and the average was determined.

### Statistical analysis

All analysis data are presented with mean and standard deviation. Data were statistically evaluated using ANOVA and Tukey Comparison Test in SPSS 26.0 (Chicago, IL, USA). *p* < 0.05 indicates a statistically significant difference.

## Results and discussion

### Production of silver nanoparticles at different temperature and pH conditions

**Fig. 3:**
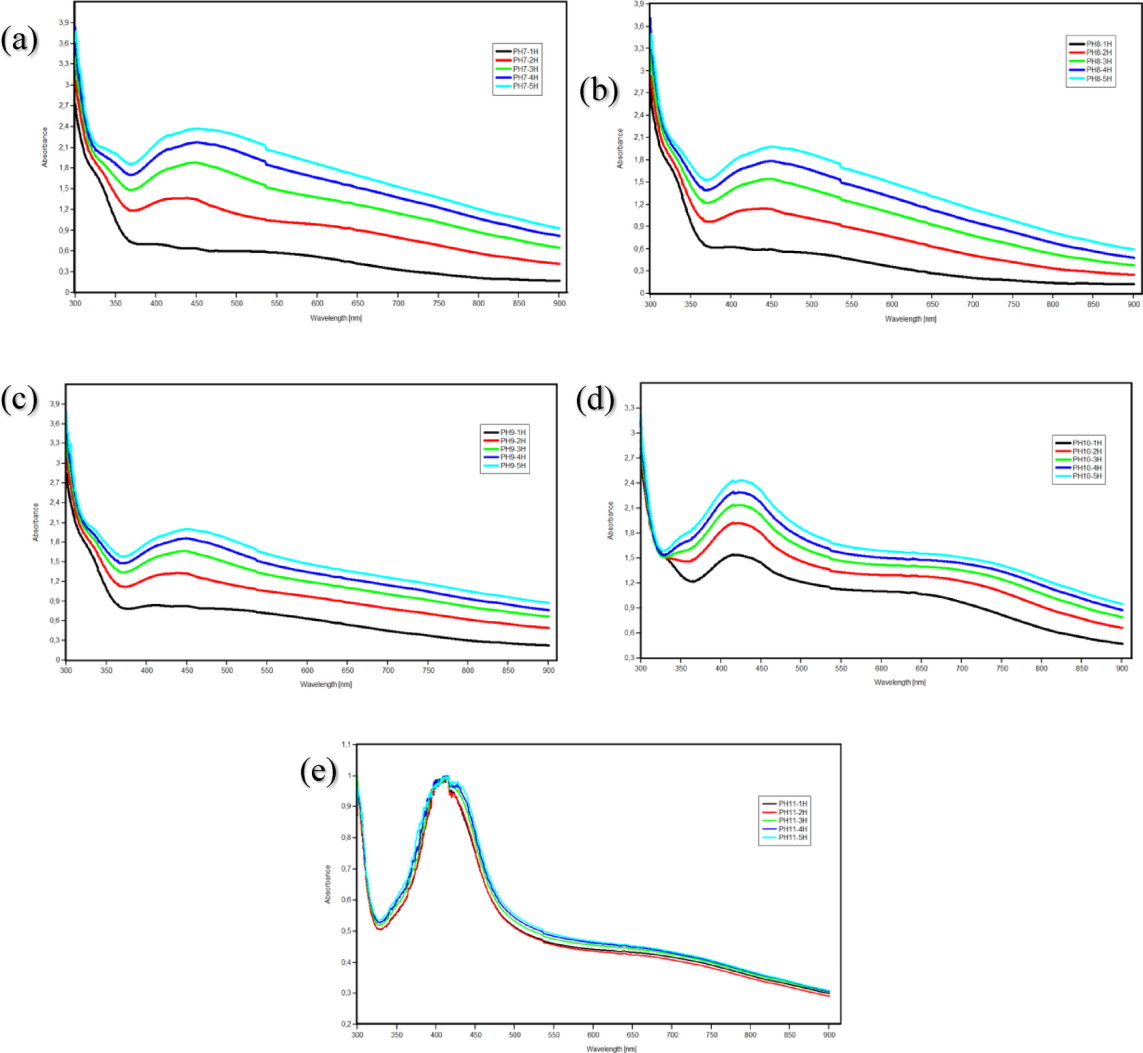
UV-Vis spectrum images of the produced AgNPs, **(a)** production pH 7 at 100 ℃, **(b)** production pH 8 at 90 ℃, **(c)** production pH 9 at 90 ℃, **(d)** production pH 10 at 50 ℃, **(e)** production pH 11 at 90 ℃.

**Fig. 4:**
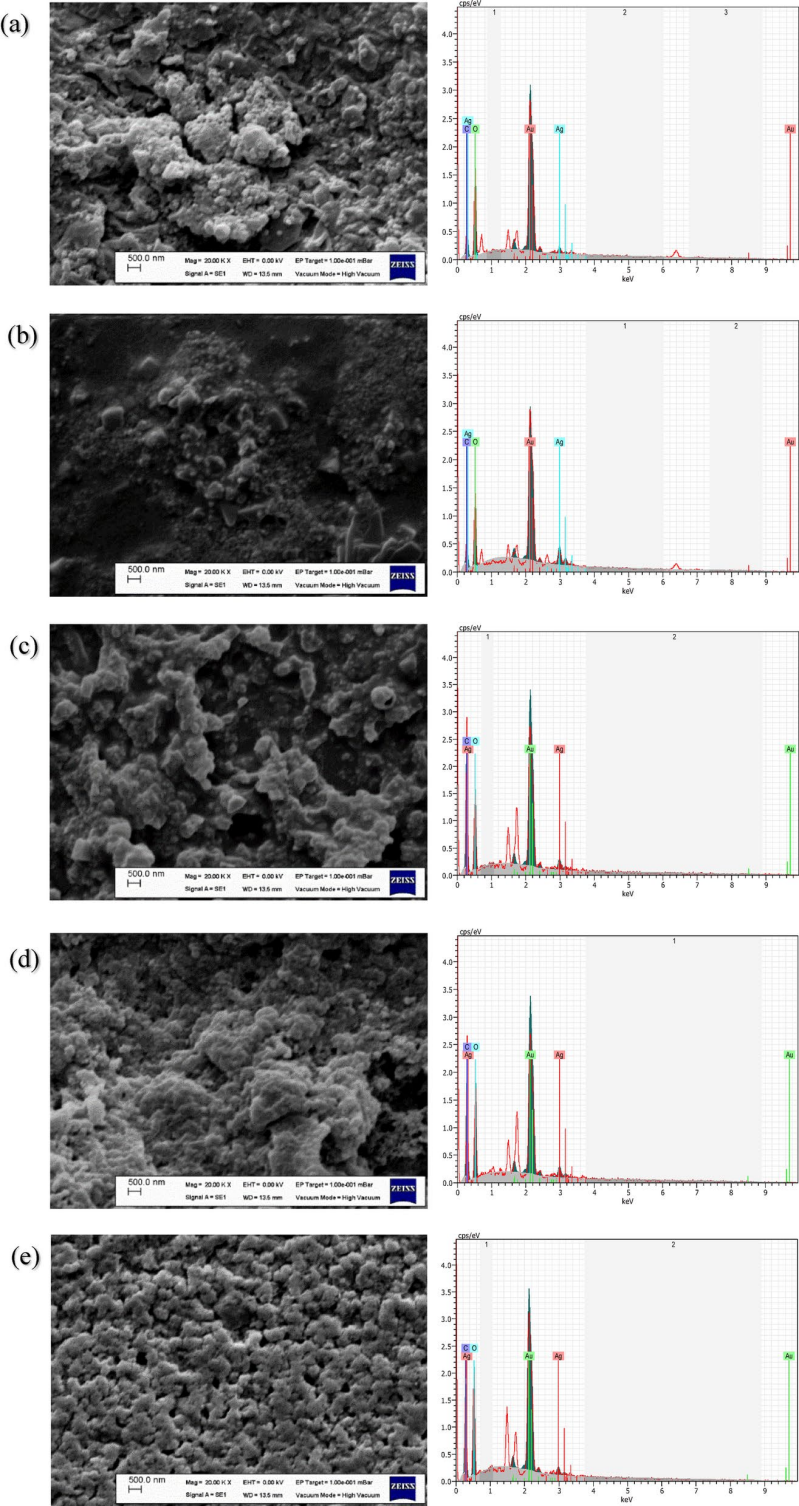
Evaluation of elemental compositions of produced AgNPs by SEM images and EDX analysis, **(a)** production pH 7 at 100 ℃, **(b)** production pH 8 at 90 ℃, **(c)** production pH 9 at 90 ℃, **(d)** production pH 10 at 50 ℃, **(e)** production pH 11 at 90 ℃.

**Fig. 5:**
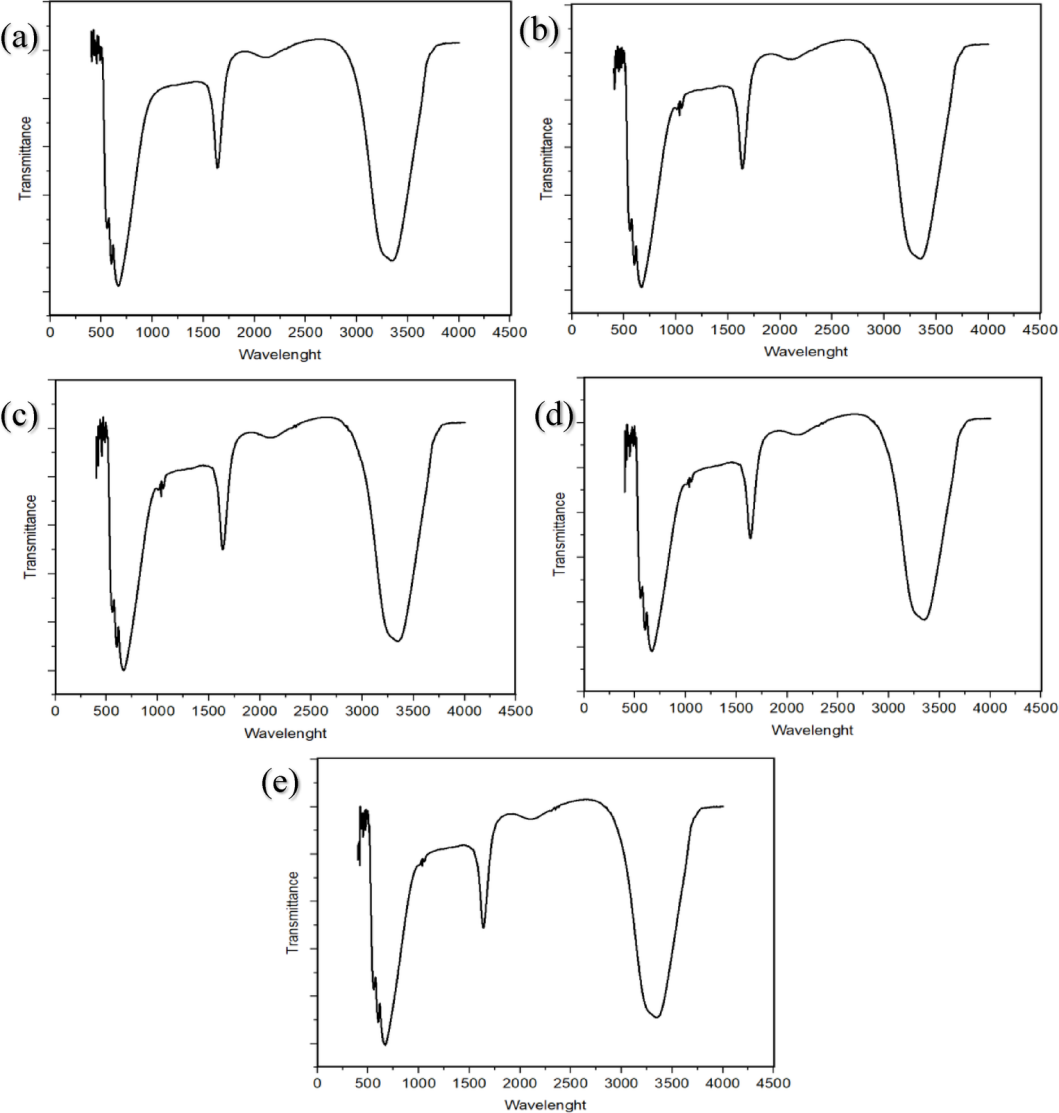
FTIR images of the produced AgNPs, **(a)** production pH 7 at 100 ℃, **(b)** production pH 8 at 90 ℃, **(c)** production pH 9 at 90 ℃, **(d)** production pH 10 at 50 ℃, **(e)** production pH 11 at 90 ℃.

**Fig. 6:**
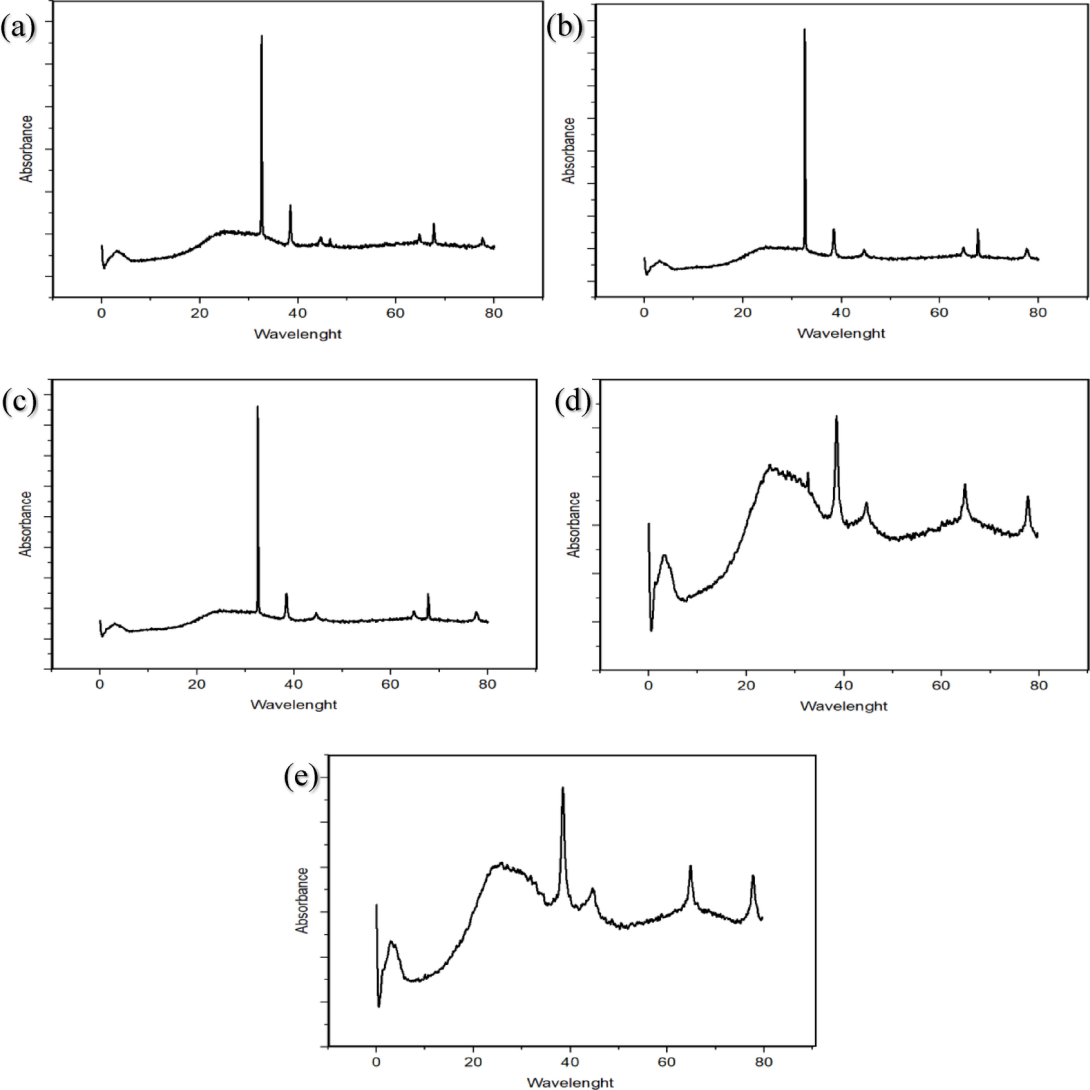
XRD images of the produced silver nanoparticles, **(a)** production pH 7 at 100 ℃, **(b)** production pH 8 at 90 ℃, **(c)** production pH 9 at 90 ℃, **(d)** production pH 10 at 50 ℃, **(e)** production pH 11 at 90 ℃.

*Viburnum opulus* extract was prepared by waiting for a certain period in a shaking incubator at room temperature. The obtained extract was used in nanoparticle synthesis. After synthesizing silver nanoparticles produced according to the specified procedure, no color change was observed in solutions at certain temperatures and pH levels. The formation kinetics of silver nanoparticles in *Viburnum opulus* extract were examined to determine the most suitable synthesis conditions. Therefore, pH levels of 7, 8, 9, 10, and 11 and temperature levels of 50, 90, and 100 °C were selected for our study. AgNPs synthesized in the chosen temperature range were centrifuged at 10,000 rpm for 10 min after the reaction. Then, they were washed with pure water. Figures (3–6) shows the images obtained during the formation. The effect of optimum pH and temperature values ​​(pH in the range of 7–11; temperature 50–100 °C) was studied to reduce silver ions for green synthesized AgNPs in 10 ml of fruit pulp. AgNPs produced under different conditions from Gilaburu fruit pulp exhibited a reddish-brown color when suspended in water. Along with the color change, the electronic energy was reduced to Ag⁰^59^.

In studies on silver nanoparticles produced by the green synthesis approach using different plant extracts, researchers have generally indicated the effects of experimental conditions on the size and composition of the produced AgNPs. Furthermore, the kinetics and mechanism of plant silver nanoparticle production continue to attract interest^12,62–64^.

Mohammadlou and other researchers have proven that silver nanoparticles produced from plant extracts are rapidly formed at ambient temperature^50^. Normally, green synthesis of AgNPs is performed at room temperature^65^, but increasing the temperature (30–90 ℃) increases the synthesis rate of AgNPs^66,67^ and also promotes the small size of AgNPs^68,69^. Makarov and other researchers and Vadlapudi and other researchers also determined that the nucleation rate of AgNPs increases with increasing temperature^70,71^. According to Khalil et al., increasing the reaction temperature caused a faster reduction of silver ions, which led to the formation of smaller-sized, homogeneously nucleated AgNPs^71^. Aziz et al. reported that the size of AgNPs may vary depending on different pH, temperature, and reaction time^72^. Patra and other researchers reported that the synthesis of AgNPs required a temperature lower than 100 ℃, which changed the properties of the formed nanoparticle^73,74^. Chitra et al. found that during AgNP synthesis at the same temperature, the solution color was yellowish and slightly brown at pH 5 and dark brown at pH 9 and confirmed that more silver nanoparticles were produced at pH 9^75^. Amaladhas et al. found that while synthesizing silver nanoparticles with *C. angustifolia* extract, no color was produced at pH 2 production, light brown at pH 4 production and dark brown at pH 11 production^76^. The temperature and pH values ​​we chose for AgNP production in the study coincide with the given references.

### Ultraviolet-visible spectrophotometry (UV–Vis)

UV-Vis spectroscopy is a widely preferred and reliable analysis method for the initial evaluation of the synthesis process of silver nanoparticles (AgNP)^77^. In the measurements made with this technique, a characteristic absorption band is observed, indicating the formation of AgNP^54,77^. The position of this band in the wavelength is affected by various factors such as the dielectric properties of the medium used, its chemical composition and the dimensions of the nanoparticles^78^. Increases in nanoparticle density and size can cause significant changes in both the absorption peak and peak height in the spectrum^77^. Synthesis of AgNP is confirmed by visual observation of a brownish color resulting from the interaction of *Viburnum opulus* plant extract with a silver metal salt. A surface plasmon resonance peak is displayed in the transmission band of the synthesized NPs which UV-Vis spectrophotometers can easily detect. This peak usually appears in the wavelength range of 400–500 nm^55,56^. In the initial stages of the synthesis, the reduction of Ag ions in Ag⁺ aqueous solution was observed by UV-Vis due to the bioactive components present *in Viburnum opulus* extract; for UV spectroscopy, 1 mM solution with five different pH degrees was used in the preparation of the AgNP preparation produced with *Viburnum opulus* extract. Ag solution was used. The UV-Vis spectra of the silver nanoparticles formed after the reaction is completed have maximum absorption at 420 to 460 nm^79^. The optimum UV-Vis spectrum images obtained from the analyses are shown in Fig. 3.

UV spectrum absorbance measurements for each sample were made every hour for 1 h, 2 h, 3 h, 4 h, and 5 h. It was observed that the peak wavelengths increased continuously as the reaction progressed, which means that nanoparticles were synthesized. The 452 nm wavelength value for the pH 7 (100 ℃) AgNP solution was recorded as the highest peak in the measurements. The 451 nm wavelength value for the pH 8 (90 ℃) AgNP solution was recorded as the highest peak in the measurements. The 450 nm wavelength value for the pH 9 (90 ℃) AgNP solution was recorded as the highest peak in the measurements. The 425 nm wavelength value for the pH 10 (50 ℃) AgNP solution was recorded as the highest peak in the measurements. The 415 nm wavelength value for the pH 11 (90 ℃) AgNP solution was recorded as the highest peak in the measurements. The UV- Vis spectra were examined, and a decrease in the spectrum wavelength values was observed due to the increase in pH value. Additionally, detailed data regarding UV-Vis spectra are given in Table 1. According to Banerjee et al., wavelength measurements at pH 7, 8, 9, and 10 show maximum absorbance, while measurement at pH 11 does not coincide with this interpretation^79^.

**Table 1:** pH and time dependent changes in UV-Vis spectral properties of Gilaburu nanoparticles.

Time (h)	pH 7		pH 8		pH 9		pH 10		pH 11	
	λmax (nm)	A	λmax (nm)	A	λmax (nm)	A	λmax (nm)	A	λmax (nm)	A
1	397	0.70	450	0.59	449	0.82	419	1.53	414	3.72
2	438	1.37	450	1.14	449	1.32	421	1.91	414	3.79
3	449	1.88	450	1.55	449	1.66	424	2.14	412	3.80
4	451	2.17	450	1.79	450	1.86	424	2.28	416	3.85
5	452	2.32	451	1.98	450	1.99	425	2.41	415	3.87

The peak values ​​of the Uv-vis spectrum of silver nanoparticles produced with *Viburnum opulus* extract were measured as 420 nm at 0 min, 442 nm at 30 min, and 450 nm after 1 h, and it was determined that the wavelength continuously increased as the reaction time progressed. This means that nanoparticles were synthesized^80^. The UV-Vis spectrum of the produced silver nanoparticles exhibited a maximum value at 413 nm^65,66^. Zia and other researchers synthesized AgNPs using *Cydonia* seed extract at 50℃, 70℃, and 90℃ and reported the UV-Vis spectrum values ​​as 462 nm, 432 nm, and 421 nm^81^. Veerasamy et al. produced AgNPs using *Mangosteen* at 7 different temperatures: 37℃, 45℃, 50℃, 65℃, 75℃, 80℃ and 90℃. The UV-Vis spectrum value was detected as 438 nm at 75 C^82^. The wavelength values ​​at the highest peaks obtained in UV-Vis measurements of the AgNPs we produced overlap with previous studies.

### Scanning electron microscopy (SEM) with EDX analysis [SEM evaluation of AgNPs

SEM evaluation is typically used to determine the size of the produced nanomaterials and predict their morphological properties^57,58^.

Many important and distinct factors are needed for the synthesis of AgNPs. Factors such as concentration, reaction time, temperature, and pH of the reacting substances affect the synthesis size, rate, and shape of NPs. These factors can be modified to regulate the overall morphology, applicability, effectiveness, shape, and size of the NPs. An SEM device obtains good-resolution images that provide morphological information about AgNPs. Scanning Electron Microscope produces images when electron beams scan the sample, examining the topography of the material and verifying its structure and elemental composition^83^. EDX is performed in conjunction with SEM^84^. High-resolution images of AgNPs produced by SEM were obtained. Spherical shapes were seen densely in the SEM image obtained from AgNPs.

Dried nanoparticles obtained at different temperatures and pH degrees were used for SEM analysis. The presence of these nanoparticles was visualized at 500 nm magnification. EDX data reveal the purity and exact chemical structure of silver nanoparticles produced by *Viburnum opulus* extract. SEM images are generally spherical, as shown in Fig. 4. Morphological differences in the figures can be attributed to the biomolecules obtained from *Viburnum opulus* extract, which serves as a capping surface. The clustering of most nanoparticles may be due to the interaction of concentrated AgNPs with biomolecules used in biosynthesis, which confirms the results obtained from previous studies^57,58,85,86^.

In addition, the elemental compositions of the AgNP produced by EDX analysis were evaluated. The EDX spectrometers in the images below revealed the elemental silver signal, indicating silver nanoparticles’ formation. The intensity of the observed peak confirmed silver nanoparticles. Surface Plasmon Resonance is responsible for this sharp silver peak^87^. These data show silver, oxygen, and carbon elements cause the peaks. Also, no peaks indicating the presence of impurities were recorded. Şahin et al., the SEM image produced with *Viburnum opulus* fruit extract showed that the synthesized AgNPs were predominantly spherical^80^.

For pH 7, the percentage of silver (% W) was determined as 36.49%, and the percentage of oxygen and carbon (% W) as 15.29% and 15.55%, respectively. For pH 8, the percentage of silver (% W) was determined as 51.93%, and the percentage of oxygen and carbon (% W) as 3.52% and 20.29%, respectively. For pH 9, the percentage of silver (% W) was determined as 45.98%, and the percentage of oxygen and carbon (% W) as 7.67% and 12.23%, respectively. For pH 10, the percentage of silver (% W) was determined as 19.97%, and the percentage of oxygen and carbon (% W) as 15.77% and 41.05%, respectively. For pH 11, the percentage of silver (% W) was determined as 66.93%, and the percentage of oxygen and carbon (% W) as 1.81% and 6.63%, respectively. According to the data obtained, the percentage of silver reached the highest value with 66.93% in pH 11-degree production, while the lowest value was 19.97% in pH 10-degree production.

In a similar study, Sharmin et al. revealed the elemental silver signal related to the presence of silver nanoparticles with EDX spectrometry. From the data obtained in the energy distribution analysis of the resulting x-rays (EDX), they saw that the peaks were due to the presence of elements such as Ag, O, and C. In addition, no peaks were recorded symbolizing the presence of foreign substances. The weight% of silver (%W) was found to be 89.31%, and the weight% of oxygen and carbon (%W) was found to be 6.77% and 3.92%^88^.

###  Fourier transform infrared spectroscopy (F-TIR)

FTIR is a method used to investigate the activity of phytoconstituents such as phenolic acids, alkaloids, proteins, terpenoids, and flavonoids, effectively reducing and stabilizing AgNPs. Functional groups (–OH, –NH2, and –CHO) present in plant extracts are oxidized by silver ions. The shift in the bands determines the reduction of the metal and the synthesis of NPs^89,90^. To identify some groups responsible for capping and stabilizing Ag nanoparticles, FTIR analysis was performed in the scope of 600–4000 cmˉ¹^59,60^.

Silver (I) ions in *Viburnum opulus* extract first bind to -OH groups of flavonoids and then reduce to Ag° to form Ag nanoparticles^91^. After the reduction reaction, surrounding molecules interact with the bonds present in the aromatic rings of silver. Thus, they stabilize Ag nanoparticles^92–94^. Different FTIR measurements were recorded under various conditions to identify the biomolecules involved in the effective stabilization and capping of AgNPs in *Viburnum opulus* extract. The FTIR spectra of AgNP colloidal solutions showed absorption bands in regions that varied depending on temperature and pH degrees.

Considering the FTIR images of silver nanoparticles produced from *V. opulus* fruit extract under different conditions; FTIR bands for pH 7 (100 °C) were determined at 3830, 3331, 2580, 2123, 1635, and 1276 cmˉ¹. FTIR bands for pH 8 (90 °C) were determined at 3851, 3322, 2117, 1635, 1303, 1053 and 1033 cmˉ¹. FTIR bands for pH 9 (90 °C) were determined at 3851, 3354, 2536, 2173, 1635, 1053 and 1033 cmˉ¹. For pH 10 (50 °C), FTIR bands were determined at 3817, 3346, 2547, 2112, 1637, 1053 and 1033 cmˉ¹. For pH 11 (90 °C), FTIR bands were determined at 3871, 3371, 2515, 2135, 1637, 1053 and 1033 cmˉ¹2. The obtained images are given in Fig. 5.

According to Şahin et al., the FTIR spectrum bands of AgNPs produced from *V. opulus* were found at 3420, 2918, 2850, 2149, 2019, 1687, 1456 and 1029 cm^− 1^. The band at 3420 cm^− 1^ represents the stretching vibration in H-bonded alcohols and phenols^95^. According to Moldovan et al., the FTIR spectrum of *Viburnum opulus* extract showed bands at the following points: 3398, 2928, 1733, 1637, and 1236 cmˉ¹^96^. Previous studies indicate that functional groups such as hydroxyl, carbonyl, aldehyde, and carboxylic acid groups of flavonoids are responsible for reducing and stabilizing silver nanoparticles^64,97^.

The bands in the range of 3322–3371 cmˉ¹ correspond to the hydroxyl found in H-bonded alcohols and phenols^61,98^. The bands observed in the range of 2515–2547 cmˉ¹ are due to saturated alkanes (-CH)^98^. The peaks observed between 2019 cmˉ¹ and 2149 cmˉ¹ indicate peptide bonds^99^. The bands in the range of 1635–1637 cmˉ¹ C = C represent vibrational aromatic and aliphatic groups^96^. The bands observed between 1053 and 1033 cmˉ¹ represent C-H/fingerprint (skeletal vibrations)^86^. The bands observed in the range of 1303 − 1276 cmˉ¹ represent the vibrational carbonyl group^100^. When the FTIR spectra of AgNPs were compared, the absorption bands of the groups were shifted. This shift is thought to be due to different pH levels and temperatures during production.

### X-ray diffraction (XRD)

The crystal structures of silver nanoparticles produced under different conditions were confirmed by X-ray diffraction diagrams. Validation of crystallite sizes of silver nanoparticles produced with *Viburnum opulus* extract was carried out using the Debye-Scherrer equation used by Gnanajobitha and other researchers^101^. Visual representations of the X-ray diffraction spectra are shown in Fig. 6.

When the XRD image of silver nanoparticles produced at pH 7 degrees and 100 °C from *V. opulus* fruit extract was examined, four Bragg reflection peaks corresponding to the planes of the cubic structure (111), (200), (220), and (311) were observed at 38.46°, 44.66°, 67.73°, and 77.66° 2θ values. The particle dimension was determined as 23.01 nm. When the XRD image of silver nanoparticles produced at pH 8 degrees and 90 °C from *V. opulus* fruit extract was examined, four Bragg reflection peaks corresponding to the planes of the cubic structure (111), (200), (220), and (311) were observed at 38.46°, 44.61°, 67.73°, and 77.66° 2θ values. The particle dimension was determined as 20.25 nm. When the XRD image of silver nanoparticles produced at pH 9 degrees and 90 °C from *V. opulus* fruit extract was examined, four Bragg reflection peaks corresponding to the planes of the cubic structure (111), (200), (220), and (311) were observed at 38.46°, 44.31°, 67.73°, and 77.66° 2θ values. The particle dimension was determined as 20.52 nm. When the XRD image of silver nanoparticles produced at pH 10 degrees and 50 °C from *V. opulus* fruit extract was examined, four Bragg reflection peaks corresponding to the planes of the cubic structure (111), (200), (220), and (311) were observed at 38.48°, 44.52, 64.84°, and 77.71° 2θ values. The particle dimension was determined as 25.24 nm. When the XRD image of silver nanoparticles produced at pH 11 degrees and 90 °C from *V. opulus* fruit extract was examined, four Bragg reflection peaks were observed at 38.43°, 44.57°, 64.84°, and 77.76° 2θ values. The particle dimension was determined as 24.74 nm. When the XRD graphs of the produced AgNPs were examined, a crystal structure with an FCC structure emerged in the production at pH 7, 8, and 9, but a semi-crystalline structure emerged in the production at pH 10 and 11. Although the structures were not fully crystalline, the particle sizes were similar to the particle sizes in the crystal structures.

Rashidipour et al. tried the synthesis of AgNPs with olive leaf extracts at five different temperatures. Then 45 °C was selected for the synthesis and the dimension of silver nanoparticles was determined as 90 nm^20^. The synthesis was performed at two different temperatures, room temperature and 80 °C. The dimension of silver nanoparticles was determined to be 7 to 22 nm at 80 °C and 6 to 24 nm at room temperature^102^.

Four Bragg reflection peaks corresponding to the characteristic planes of the face-centered cubic structure (111), (200), (220), and (311) were detected at 38.44°, 44.66°, 65.01°, and 78.21°, and the particle size was determined as 52.32 nm^80^. XRD proved synthesized AgNPs. Four different diffraction spots corresponding to the (111), (200), (222), and (311) cubic structures of metallic silver were observed at the angles of 38.38°, 44.52°, 64.58°, and 82.46°, respectively^90^. AgNP produced from *Viburnum opulus* showed five Bragg reflection peaks at 38.25°, 44.35°, 64.73°, 77.36° and 82.33° corresponding to the planes of (111), (200), (220), (311) and (222) face-centered cubic structure^96^.

### Evaluation of antimicrobial activity degree of synthesized AgNPs

**Fig. 7:**
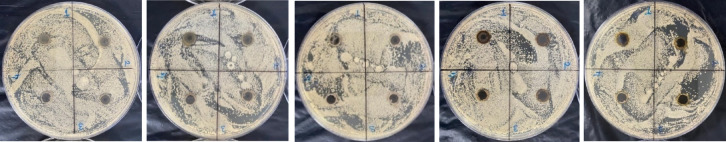
Antimicrobial activity of *Viburnum opulus* silver nanoparticles on *C. albicans*.

**Fig. 8:**
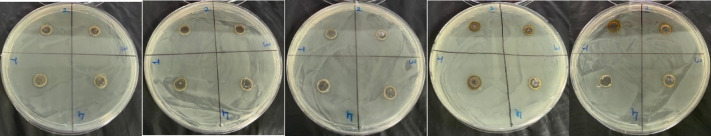
Antimicrobial activity of *Viburnum opulus* silver nanoparticles on *E. coli*.

**Fig. 9:**
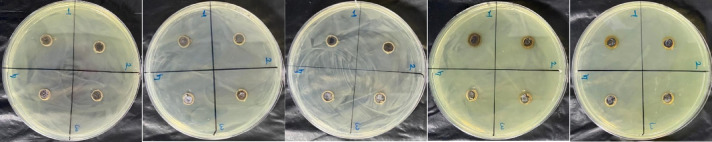
Antimicrobial activity of *Viburnum opulus* silver nanoparticles on *P. aeruginosa*.

**Fig. 10:**
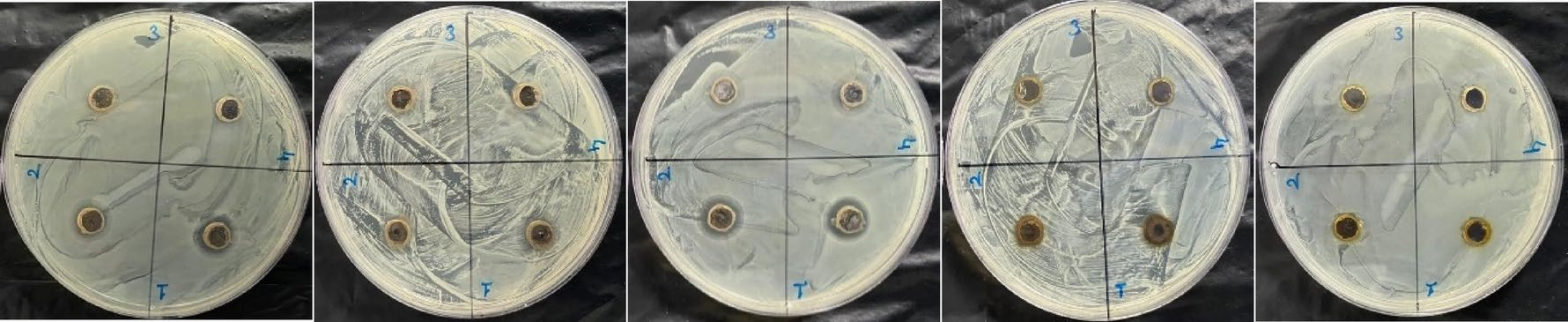
Antimicrobial activity of *Viburnum opulus* silver nanoparticles on *S. aureus*.

**Fig. 11:**
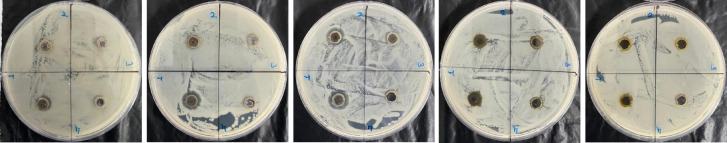
Antimicrobial activity of *Viburnum opulus* silver nanoparticles on *S. pyogenes*.

Antimicrobial activities of synthesized *Viburnum opulus* AgNPs were evaluated toward Gram-positive bacterial species (*S. pyogenes* and *S. aureus*), Gram-negative bacterial species (*P. aeruginosa* and *E. coli*), and *C. albicans* using agar diffusion method at different sample concentrations (50, 100, 200, 400 µg/mL). The average diameters of inhibitory zones were measured at the end of the incubation process, and the results were provided in Table 2^103^. AgNPs could stop the growth of the three pathological bacteria examined but did not show antibacterial activity against *P. aeruginosa* or antimicrobial activity against *C. albicans*. Figure (7–11) shows images obtained from the analysis.

**Table 2:** At different temperatures and pH degrees antibacterial activity of the produced silver nanoparticle against various pathogens.

PH degrees	Concentration	Pathogenic Microorganisms				
	(µg/ml)	*E. coli*	*S. aureus*	*S. pyogenes*	*P. aeruginosa*	*C. albicans*
pH 7	50	10.13 ± 0.17^ay1^	14.13 ± 0.09^ax2^	10.17 ± 0.12^ay3^	-	-
	100	9.10 ± 0.08^by1^	13.07 ± 0.05^bx2^	7.10 ± 0.08^bz4^	-	-
	200	9.13 ± 0.09^by1^	10.10 ± 0.08^cx2^	-	-	-
	400	-	9.07 ± 0.05^dy2^	7.03 ± 0.05^by2^	-	-
pH 8	50	10.13 ± 0.12^az1^	14.07 ± 0.05^ax2^	13.17 ± 0.12^ay2^	-	-
	100	9.10 ± 0.08^by1^	10.13 ± 0.09^bx3^	8.10 ± 0.08^bz3^	-	-
	200	-	-	-	-	-
	400	-	-	-	-	-
pH 9	50	10.13 ± 0.09^az1^	15.20 ± 0.14^ax1^	14.13 ± 0.12^ay1^	-	-
	100	9.13 ± 0.09^bz1^	14.10 ± 0.08^bx1^	13.07 ± 0.09^by1^	-	-
	200	9.03 ± 0.05^bz1^	14.03 ± 0.05^bx1^	10.07 ± 0.05^cy1^	-	-
	400	-	13.07 ± 0.09^cx1^	8.07 ± 0.09^dy1^	-	-
pH 10	50	-	-	10.17 ± 0.12^ax3^	-	-
	100	-	8.13 ± 0.09^by4^	9.13 ± 0.12^bx2^	-	-
	200	8.10 ± 0.08^ay2^	8.10 ± 0.08^by3^	9.13 ± 0.09^bx2^	-	-
	400	8.03 ± 0.05^ay1^	9.03 ± 0.05^ax2^	8.07 ± 0.05^cy1^	-	-
pH 11	50	-	8.07 ± 0.09^ax3^	8.07 ± 0.09^ax4^	-	-
	100	-	8.03 ± 0.05^ax4^	-	-	-
	200	-	7.13 ± 0.09^bx4^	-	-	-
	400	8.10 ± 0.08^ax1^	7.03 ± 0.05^by3^	-	-	-

( - ) Could not be detected.

The data are expressed as mean ± standard deviation.

Different letters (a-d) in the same column indicate the effect of various concentrations.

Different letters (x-z) in the same column indicate the effect of various microorganisms.

Different numbers (1–4) in the same column indicate the effect of various concentrations.

Table 2 shows the effects of AgNPs produced at different pH and temperature levels on pathogenic microorganisms. When the impact on microorganisms was examined, the best zone diameter was calculated as 15.20 ± 0.14 mm at a concentration of 50 µg/µl in pH 9 production on *S. aureus*. The smallest zone diameter was calculated as 7.03 ± 0.05 mm at a concentration of 400 µg/µl in pH 11 production on *S. aureus* and 7.03 ± 0.05 mm at a concentration of 400 µg/µl in pH 7 production on *S. pyogenes*. When the effects of different concentration values ​​of 100 µg/µl and 200 µg/µl on *S. aureus* microorganism in pH 9 production were examined, no statistically significant difference was observed in zone diameters even though the concentration increased (*p* < 0.05). No antimicrobial effect was observed on *Pseudomonas aeruginosa* and *Candida albicans*. Researchers suggest that the antimicrobial activity of AgNPs against microbes is due to the K⁺ ions produced by bacteria. Bacterial plasma or cytoplasmic membrane can bind to silver nanoparticles by combining with critical enzymes and DNA. Cell walls are disrupted after vacuoles are exposed to silver ions^104^. Reactive oxygen species (ROS) are produced and the membrane is damaged^105^. According to previous studies, the mechanism that causes the expansion of the inhibitory region is due to the increase in the proton mobility force on the bacterial surface due to the formation of ionic bonds with AgNPs^106^. In addition, Ag ion distribution from silver nanoparticles is a prominent feature of their antibacterial activity. Silver ions can form thiol linkages in enzymes for bacterial biological processes and cellular functions. This event destroys the enzymatic action and leads to bacterial death^107^.

Pal and colleagues found that the shape of nanoparticles can change antimicrobial activity^108^. Sondi and other researchers characterized the antimicrobial activities of AgNPs as opposed to *E. coli*^109^. Researchers also found that the formation of pits in the bacterial cell wall and the accumulation of AgNPs in the cell membrane help in increasing the permeability of AgNPs and eventually killing the bacterial cell^109,110^. Antibacterial trials performed by disk diffusion method showed that cold ethanol extract of fresh fruit inhibited *Streptococcus pyogenes* and hot water extract of dried fruit inhibited *Streptococcus pyogenes*,* Staphylococcus aureus*, and *Staphylococcus epidermidis*. Again, in a similar study, the inhibitory effects of *V. opulus* extracts on *Staphylococcus aureus* and *S. epidermidis* were determined^111^. Česoniene et al., found that *V. opulus* fruit extracts had antimicrobial effects against both Gram (+) and Gram (-) bacteria, but had no effect against yeasts^42^.Many researchers have stated that AgNPs produced in smaller sizes have more activity owing to their raised surface region^112^. Shamelie et al. reported in their research that the antibacterial activities of AgNPs in polyethylene glycol can be changed by controlling the size of the nanoparticles as the activity of AgNPs decreases with larger particle size^113^. Much research has been carried out to determine the antifungal effects of silver nanoparticles. Kim and other researchers reported that silver nanoparticles have antifungal effects on *Candida albicans*^114^. Gajbhiye and other researchers demonstrated the activity of synthesized AgNPs on *Candida albicans*,* Fusarium semitectum*,* Phoma glomerata*,* and Pleospora herbarum*^115^. The antifungal effect of silver nanoparticles is thought to be due to disruption of membrane integrity and inhibition of budding^98^. In the antimicrobial analysis of *V. opulus* AgNP production against various microorganisms, zone diameters were found to be between 6.05 and 7.15 mm^80^.

## Discussion

This study synthesized AgNPs under different temperatures and pH conditions with an extract from Gilaburu (*V. opulus*) fruit. UV-Vis, SEM-EDX, FTIR, and XRD methods were used to analyze the AgNPs formed due to the synthesis. In addition, their antimicrobial properties against many microorganisms were investigated extensively. AgNPs produced from *Viburnum opulus* fruit showed significant antibacterial effects against *S. aureus*,* S. pyogenes*, and *E. coli*.

As a result of the characterization processes, when the UV-Vis graphs of biosynthetic silver nanoparticles (AgNPs) were examined, a decrease in spectral wavelength values ​​was observed due to the increase in pH value. According to Banerjee et al., measurements made at pH 7, 8, 9, and 10 showed maximum absorbance, while those made at pH 11 were not in the maximum absorbance range. In SEM analysis, it was determined that AgNPs dispersed under the influence of the reaction temperature. In addition, the elemental compositions of the produced AgNP were evaluated using EDX analysis. According to the obtained data, while the weight% of silver was the highest at pH 11 production with 66.93%, the lowest value was 19.97% at pH 10 production. When XRD graphs were examined, it was determined that the smallest particle size was 20.25 nm at pH 8 production and the largest particle size was 25.24 nm at pH 10 production. They stopped the growth of the three pathogenic bacteria studied but did not show antibacterial activity against *P. aeruginosa* or antimicrobial activity against *C. albicans*.

The reaction in AgNP production usually takes place at room temperature. However, this results in an increase in the reaction time. Therefore, the reaction rate can be increased directly to the mixture temperature during the synthesis. As the reaction temperature increases, Ag⁺ ions decrease rapidly and small-sized AgNPs are obtained^116^. Therefore, the temperature values ​​in the reaction are usually determined as 90 and 100 ℃. The pH value of the solution used in the response shows significant changes in AgNP synthesis since it affects the biomolecules and changes their reducing properties^117^. The rapid production of nanoparticles at neutral pH may be due to the ionization of phenolic components found in the plant extract. In the synthesis carried out at low pH, pH promotes aggregation, which leads to the formation of large-sized nanoparticles, but in the synthesis carried out at high pH, ​​much more stable and small-sized nanoparticles are formed^118^. According to the data obtained from our study, UV-Vis showed maximum absorbance values ​​at pH 7 and pH 8. Parameters such as temperature and pH in the reaction medium affect the spectral properties of UV absorbance bands and determine the appearance of signals belonging to surface plasmon resonance. These stable and homogeneous structures interact more effectively with microorganisms and increase antimicrobial activity. SEM-EDX result was determined as 66.93% by weight at pH 11. The smallest particle sizes are 20.25 nm at pH 8 and 20.52 nm at pH 9. In addition, AgNPs produced at pH 7, pH 8, and pH 9 have higher antimicrobial effects on microorganisms. It is recommended that AgNP production from *Viburnum opulus* fruit be carried out between pH 7 and pH 8.

Green synthesis of silver nanomaterials is a cheap and environmentally friendly approach. It also hopes to provide increased biological activities due to its production in a synergistic synthesis environment containing anti-cancer, anti-bacterial, and anti-fungal activities^62^. Therefore, in this research, AgNPs were produced from the water based extract of *Viburnum opulus*, which is known for its high biological activities such as anti-inflammatory, antiseptic, antipyretic, antibacterial, and antifungal. It is well known that *Viburnum opulus* fruits have a strong antioxidant activity due to the presence of a wide variety of phytocompounds such as polyphenols, ascorbic acid, and carotenoids. Therefore, in this study, *Viburnum opulus* fruit extract was used to reduce silver ions to silver nanoparticles. Compared to physical and chemical approaches, various advantages of green synthesis of AgNPs with *Viburnum opulus* extract were identified, such as the absence of the use of hazardous chemicals, high energy, and pressure. Biosynthesized AgNPs have potential applications, especially in antimicrobial activity, food safety, and food packaging. In agriculture, the provision of nanotechnology-based NPs has yielded positive results for plant growth, nutrition, and increased resistance to plant diseases. AgNPs synthesized using nanotechnology are used in plant disease management in nanoherbicides, nanopesticides, nanofungicides, DNA, and gene delivery. *Viburnum opulus* will play a critical role by paving the way for the production of more controlled nanoparticles with desired properties and its further studies will open new horizons in the field of development. In future studies, the biological effects of the synthesized nanoparticles, such as antioxidant and anticancer, can be investigated in detail. In addition, different plant species can be used by comparing them and the surface properties and structural stability of the nanoparticles can be analyzed with advanced techniques. Possible applications in biomedical, environmental and industrial areas should also be evaluated.

## Data Availability

The datasets used and/or analyzed during the current study are available from the corresponding author upon request.
